# BGRMI: A method for inferring gene regulatory networks from time-course gene expression data and its application in breast cancer research

**DOI:** 10.1038/srep37140

**Published:** 2016-11-23

**Authors:** Luis F. Iglesias-Martinez, Walter Kolch, Tapesh Santra

**Affiliations:** 1 Systems Biology Ireland, University College Dublin, Belfield, Dublin 4, Republic of Ireland; 2 Conway Institute of Biomolecular and Biomedical Research, University College Dublin, Belfield, Dublin 4, Ireland; 3 School of Medicine and Medical Science, University College Dublin, Belfield, Dublin 4, Ireland

## Abstract

Reconstructing gene regulatory networks (GRNs) from gene expression data is a challenging problem. Existing GRN reconstruction algorithms can be broadly divided into model-free and model–based methods. Typically, model-free methods have high accuracy but are computation intensive whereas model-based methods are fast but less accurate. We propose Bayesian Gene Regulation Model Inference (BGRMI), a model-based method for inferring GRNs from time-course gene expression data. BGRMI uses a Bayesian framework to calculate the probability of different models of GRNs and a heuristic search strategy to scan the model space efficiently. Using benchmark datasets, we show that BGRMI has higher/comparable accuracy at a fraction of the computational cost of competing algorithms. Additionally, it can incorporate prior knowledge of potential gene regulation mechanisms and TF hetero-dimerization processes in the GRN reconstruction process. We incorporated existing ChIP-seq data and known protein interactions between TFs in BGRMI as sources of prior knowledge to reconstruct transcription regulatory networks of proliferating and differentiating breast cancer (BC) cells from time-course gene expression data. The reconstructed networks revealed key driver genes of proliferation and differentiation in BC cells. Some of these genes were not previously studied in the context of BC, but may have clinical relevance in BC treatment.

Cellular functions depend on the precise regulation of thousands of genes which are activated or silenced by transcription factors (TFs)^1^. The networks representing the interactions between TFs and their target genes are typically known as GRNs and can be reconstructed from temporal measurements of gene expressions^2^,^3^,^4^,^5^. The GRN reconstruction methods can be classified into two main categories; model-based and model-free methods. Model-based methods aim to capture the regulatory interactions by fitting mathematical models of gene regulation to observed gene expression data^2^,^4^,^5^. On the other hand, model-free approaches use information-theoretic criteria to infer the structure of the network^3^,^6^. Though the performances of GRN reconstruction methods depend on several aspects such as data type, network properties of the GRN etc^7^., generally, model-based methods tend to be faster but have lower predictive performance than model-free methods 2015^6^. However, model-free methods are often not scalable enough to reconstruct genome-wide GRNs^5^,^6^ in reasonable time. Typically, model-based methods formulate the expression of a gene as a function of its regulators, evaluate competing models containing different sets of regulators and chose those which closely predict the target gene expression^4^,^5^,^8^. Although vast majority of model based methods assume that the expressions of a gene and its regulators are linearly dependent^5^,^9^,^10^,^11^,^12^, these methods use different model search algorithms e.g. Least Absolute Shrinkage and Selection Operator (LASSO), Dantzig Selector, elastic net, Markov Chain Monte Carlo and Heuristic search^5^,^12^,^13^,^14^,^15^,^16^,^17^,^18^,^19^. Some of these methods such as LASSO and elastic net choose the best model, others such as MCMC or Heuristic search based Bayesian Model Averaging (BMA) methods^5^,^12^,^13^,^15^,^18^ select multiple models that provide close fits to the data and use these to estimate an average model along with its confidence interval. It is also possible to incorporate different types of existing data in BMA^5^,^12^,^18^ to increase the accuracy of the reconstructed GRN.

We developed BGRMI, a model-based method that relies on the principles of BMA for inferring GRNs from time course gene expression data. BGRMI uses discretized ordinary differential equation (DODE) based mathematical models to formulate the interactions between each gene and its regulators. It formulates the rate of change in a gene’s expression as a function of the expressions of its regulators, takes basal expression and self-regulation into account and therefore provides a more realistic model of gene regulation than many existing methods. These models are then used in a Bayesian framework to evaluate how likely a set of TFs is to regulate a certain gene. We developed a greedy heuristic search algorithm to explore different combinations of TFs and find the most likely TF combinations for each gene. The proposed algorithm is faster and more scalable than many existing methods. The average of some of the most likely models was then used to represent the regulatory model of the gene. We compared the accuracy of BGRMI against other methods using *in-silico* and *in-vivo* benchmark datasets. BGRMI consistently out-performed most of the other competing methods in our benchmarking study. We then showed how additional data sources, e.g. ChIP-seq and the protein-protein interaction (PPI) between TFs can be incorporated as prior knowledge in the core BGRMI formulation. Finally, we applied BGRMI to study the transcriptional mechanisms that lead to proliferation and differentiation in BC cells by combining ChIP-seq, PPI and time course gene expression profiles. Our study uncovered previously unknown transcriptional mechanisms that drive phenotypic changes in BC cells.

## Method

A brief overview of the BGRMI algorithm is as follows. We first developed a mathematical model of TF-mediated gene regulations. The model can predict the temporal changes in the expressions of target genes using the temporal expression patterns of TFs as input. This model is then used to evaluate different combinations of TFs to find those that closely predict target gene expressions. This is done by iteratively exploring different TF combinations, calculating the posterior probability of each of these combinations to predict target gene expression, and selecting those with high probabilities. The average of selected gene regulation models for each gene is used as its regulation model. Below we describe each step of our algorithm in detail.

### The mathematical model of gene regulation

We used a discretized form of ODEs to formulate the dependence of a target gene on its regulators as shown below.


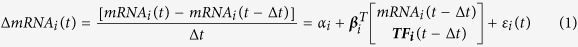


Here, *mRNA*_*i*_(*t*) is the expression of gene *i* at time *t, α*_*i*_ is the basal gene expression rate, ***β***_*i*_ is a vector that contains the coefficients of self regulation (by means of degradation, auto-activation/inhibition) and the regulation by a set of TFs (***TF***_***i***_), ***TF***_***i***_(*t* − Δ*t*) are the expressions of the TFs that regulate gene *i* at time (*t* − Δ*t*). *ε*_*i*_(*t*) is the model fitting error caused by the measurement noise in expression data. Since measurement noise is random *ε*_*i*_(*t*) is a random variable and typically has Gaussian distribution with zero mean and variance *σ*^2^, i.e. *ε*_*i*_(*t*)~*N*(0, *σ*^2^)^12^,^18^. The error variance (*σ*^2^) depends on many factors such as biological variability and measurement noise, and is typically unknown.

### The posterior probability of a gene regulation model

We used Bayesian statistics to calculate this probability. There are two main components of Bayesian formulations; (a) the prior probability (*p(M*_*k*_)) which represents how well a model (*M*_*k*_) is supported by prior knowledge, and (b) the likelihood function (*p(**mRNA***_***i***_|*M*_*k*_)) which evaluates how well a model explains experimental data. By Bayes’ rule^20^, the posterior probability (*p(M*_*k*_|***mRNA***_***i***_)) is proportional to the product of these two entities and represents how well a model (*M*_*k*_) is supported by prior knowledge and experimental data combined.

In the absence of prior knowledge, we assumed that sparse regulatory models (i.e. those involving fewer TFs) are a priori more likely than dense models (i.e. those involving a large number of TFs). This assumption was formulated by assigning the following prior distributions over regulatory models (*M*_*k*_)^5^: *p(M*_*k*_) = *L*^−2.66^, where *L* is the number of regulators in the model. However, there is a wealth of publicly available information about GRNs of several organisms such as yeast, E. coli, humans etc. This information can be used to formulate more informative priors for reconstructing GRNs of these organisms. We shall discuss the formulation of priors for human GRNs using ChIP-seq data^12^ in a later section where we describe the implementation of BGRMI on human transcriptomic data.

The likelihood of a gene regulation model (*M*_*k*_) is the probability that the observed expression pattern (***mRNA***_***i***_) of gene *i*, can be predicted by the model (*M*_*k*_), and has the following form^5^,^12^:


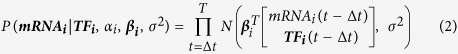


The likelihood function in Eq. 2 depends on the model parameters (*α*_*i*_, ***β***_*i*_ *σ*^2^), whose values are typically unknown. Therefore, we evaluated the average of the likelihood (Eq. 2) over all possible values of the model parameters. The average likelihood is also called the marginal likelihood (*P(**mRNA***_***i***_|*M*_*k*_)). To analytically calculate the marginal likelihood we assigned conjugate prior distributions to each of the unknown variables. These distributions represent our prior knowledge of how likely a parameter is to have a certain value. Following Fernandez *et al*.^13^ we assigned uninformative Jeffrey’s prior^13^,^21^ distribution to the basal expression rates *α*_*i*_, *p(α*_*i*_) = 1, which implies that *α*_*i*_ is equally likely to have any real value. The regulation coefficients (***β***_*i*_) were assigned Zellner’s g prior^13^,^22^.


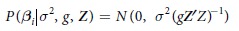



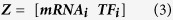


which implies that ***β***_*i*_ may have a wide range of positive and negative values depending on the Zellner’s constant *g* and error variance *σ*^2^. Note that *σ*^2^ is unknown, and therefore we assigned a non-informative Jeffrey’s prior^13^,^21^, 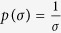 which suggests that the probability of *σ*^2^ is inversely proportional to itself, i.e. it is more likely to have smaller values than larger ones. The marginal likelihood (*P(**mRNA***_***i***_|*M*_*k*_)) is calculated by integrating the product of the likelihood and the above priors with respect to the unknown parameters and has the following form^5^,^12^.


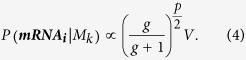



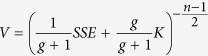



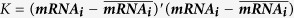


Here *p* is the number of TFs in the model *M*_*k*_, and *n* is the number of observations, SSE is the squared error between the observed expressions of gene *i* and those predicted by the model when its parameters are estimated using linear regression. The marginal likelihood in Eq. 4 depends on the Zellner’s constant g which is set to the following value recommended by Fernandez *et al*.^13^:


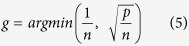


The posterior probability (*P(M*_*k*_|***mRNA***_***i***_)) of a potential regulatory model (*M*_*k*_) of gene *i*, is then calculated using the following formula:


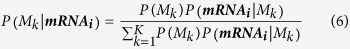


### Model Search

**Figure 1:**
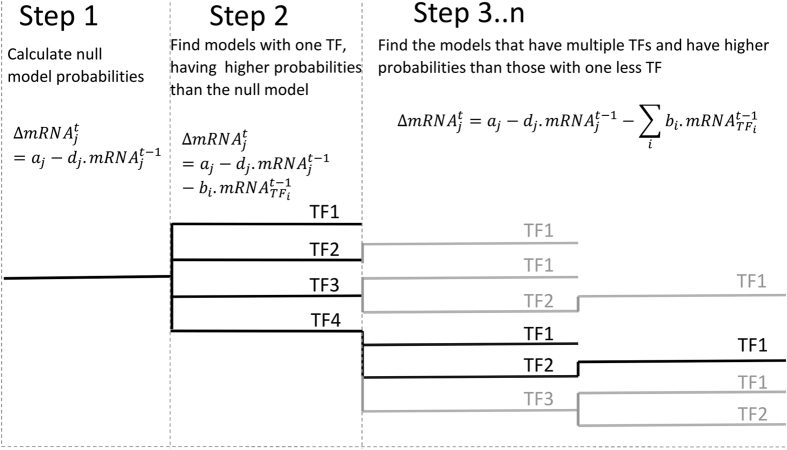
Workflow of the heuristic model search algorithm. On the first step the marginal likelihood of the null model is calculated. Then each TF is evaluated as a variable independently and only TFs whose marginal likelihood is higher than the null model’s are further expanded. The highest marginal likelihood of the single TF models is selected as a threshold or bound to evaluate the nested models with two TFs.

We developed a heuristic algorithm to search for models with high posterior probabilities. The proposed algorithm (Fig. 1) is inspired by Occam’s Up^23^ and Branch and Bound algorithms^24^. The procedure starts by evaluating the posterior probability of the null model (*M*^0^) which does not have any regulator except itself. In the next step, the null model is expanded by adding one TF. Each candidate TF is added one by one and the posterior probabilities of the new models with a single TF (*M*^1^) are evaluated. The models that have higher posterior probabilities than the null model are selected and their posterior probabilities are compared. The highest posterior probability is used as a cut-off for the next stage. The selected models are further expanded by adding a new TF. Each of the remaining TFs (the TFs other than the ones already in the model) is added one by one. The models which have higher posterior probability than the cut-off are then kept and compared, and the highest posterior probability is then selected as the new cut-off for the next stage. This process is repeated until adding a new TF does not improve the posterior probability any further. Below we provide a pseudocode for our algorithm.

### Pseudocode

***P*** ← ***0 ***_***N*****×*****N***_ ***#** Initialize probability matrix*

***For** each gene G*_*i*_

***{***

*Active* ← *1 # Flag to terminate while loop*

*Th* **← ***P(M*_*0*_) *# Non-normalized posterior probability of the null model*

***MQ*** **← ***{G*_*j*_, *j* = *1*…*N, j* ≠ *i} # Initialize Model queue which contains all genes but G*_*i*._

*NC* **← ***0 # Initialize normalization constant*

***AM*** **← **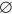 *# Initialize accepted models*.

*While* (*Active*==*1*)

 *{*

  *For each M*_*j*_ *in **MQ** # For each model in the model queue*

   *{*

    *PM*_*j*_ **← ***P(M*_*j*_) # *Calculate non normalized posterior of model M*_*j*_

    ***if** PM*_*j*_ > *Th*

       { ***AM*** 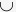 *M*_*j*_ # *Add model j to the set of accepted models*

      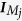 **← ***Indexes of genes in M*_*j*_

      *P(i,*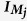) = *P(i*,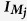) + *PM*_*j*_ # *Update the posterior of an edge*

      *NC* **← ***NC* + *PM*_*j*_; # *Update the normalization constant*

     **} #***End of if*

   **} #** *End of for*

***MQ*** **← ***All models that can be generated by extending each model in **AM** with one more gene.*

 ***if AM***==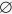 # *If **AM** is empty*

  *{*

   *Active* = 0 # *set Active to zeros*

  *}***#** *End of if*

 ***AM*** **← **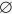 # *Empty **AM***

  ***}*** **#** *End of while*

  ***P**(i*,:) = ***P**(i*,:)/*NC*;

  **} #** *End of for*

### Model averaging

The models selected by the above algorithm are used to estimate the probability of each TF-gene interaction and its strength. The probability that a TF (*j*) regulates a gene (*i*) is the sum of the probabilities of the models which include the TF (*j*)^15^, i.e.


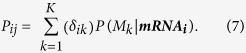


Here *K* is the number of selected models, *δ*_*ik*_ = 1 if TF *j* is part of model *k* and *δ*_*ik*_ = 0 otherwise. The interaction strength between a TF (*j*) and its target gene (*i*) is calculated by taking weighted average of its expected value in each selected model (*M*_*k*_), the weight being the posterior probability *P(M*_*k*_|***mRNA***_***i***_) of the model (*M*_*k*_)^15^


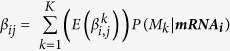



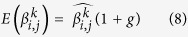


Here 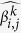 is the maximum likelihood estimate of the regulation coefficient of the TF *j* on gene *i* in model *M*_*k*_. If *β*_*ij*_ is positive then we assume that the TF *j* is an activator of gene *i* and if it is negative, the opposite is true.

## Results

We first evaluated BGRMI’s accuracy on several *in silico* and *in vivo* benchmark datasets and compared its performance with other algorithms. Then we applied BGRMI to study human BC transcription regulatory network. Below we discuss the results of our analysis in detail.

### The DREAM4 *In Silico* Network Inference Challenge dataset

The DREAM4 *In Silico* Network Challenge contains ten *in silico* GRNs, five of which consist of *10* genes each and the remaining five have *100* genes each. The dynamics of each of these networks in response to a series of perturbations were simulated and the resulting time course gene expression profiles were published by the DREAM consortium^6^ for benchmarking network inference methods. We used BGRMI to analyse these data and calculate the probabilities and strengths of all possible interactions in each of these networks. The interactions which have higher probabilities than a pre-determined threshold constitute the reconstructed GRNs. The accuracy of the reconstructed GRNs is estimated by comparing these with the gold-standard networks and is dependent on the choice of the threshold probability. We used Precision Recall (PR) curve^25^ to estimate these accuracies in an unbiased manner, independently of particular choices of threshold probabilities. PR curve is calculated by gradually increasing the threshold probability from 0 to 1, and for each threshold, calculating the precision and recall of the GRN reconstructed at that threshold^25^. Precision and recall are the ratios of the numbers of correctly inferred interactions vs all interactions in the reconstructed and the gold standard networks respectively^25^. The Area under the PR curve (AUPR) provides an unbiased scalar estimate of the accuracies of the reconstructed GRNs^5^,^6^. The AUPR values of the 10 and 100 gene networks reconstructed by the BGRMI algorithm are provided in Table 1. For comparison, we have also provided the AUPR values of the networks reconstructed by several other state-of-the-art algorithms, e.g. Jump3 the lagged time variant of GENIE3^3^, CLR^26^, Inferelator^4^, G1DBN^27^, and ScanBMA^5^, which also claimed to have performed very well on the same datasets. BGRMI consistently performed well, achieving the highest AUPRs in 4 out of 10 networks (2 each of the 10 and 100 genes networks). It also achieved the highest average AUPR (0.401) across all ten datasets (Table 1), a noticeable improvement over its closest competitor Inferelator (avg. AUPR = 0.3605 across all ten datasets.)

### *In Vivo* benchmark data

To further test BGRMI we used time course gene expression data from a synthetic GRN, called *In vivo* Reverse-engineering and Modeling Assessment (IRMA) network, which was purposefully built to assess the performances of network reconstruction methods^28^. The IRMA network was synthesized in the yeast *Saccharomyces cerevisiae*. The network has 5 genes and 6 regulatory interactions and can be switched on or off by culturing cells in galactose or glucose, respectively. The expression levels of the genes in the network were measured using quantitative RT-PCR at different time points in two different sets of experiments. In the first set, cells were stimulated with galactose and the network was switched on, whereas in the second set the network was switched off by adding glucose.

Table 2 shows the AUPRs of the GRNs reconstructed by all methods which were used for performance comparison of in-silico data. Additionally, we added the performance of the TSNI algorithm^29^ which was originally used to reconstruct the IRMA network^28^. BGRMI had the highest accuracy for the Switch-On dataset by a large margin. However, on the Switch-Off dataset, Jump3 performed the best. These results suggest BGRMI performs well, not only on in-silico datasets but also on *in-vivo* experimental data.

### Execution time of BGRMI

We measured the execution time of our method on the DREAM4 and IRMA networks. We used a 32-GB RAM, 1.7 GHz Intel core i7 computer. The results are summarized in Table 3.

### Uncovering transcriptional mechanism governing proliferation and differentiation in BC cells

**Figure 2:**
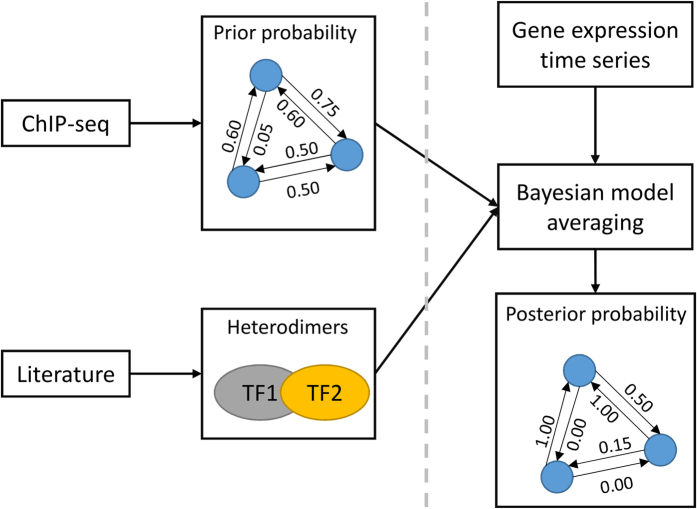
Workflow of BGRMI implementation on time course gene expression profiles on human BC cells.

Several types of BCs are formed when breast tissue cells stop differentiating and keep proliferating^30^. Therefore, it is important to determine the molecular mechanisms that govern proliferation and differentiation in these cells. For this purpose, Mina *et al*.^31^ measured time course gene expression profiles of MCF-7 BC cells after artificially inducing proliferation and differentiation by stimulating these cells with Heregulin (HRG) and Epidermal Growth Factors (EGF), respectively^31^. We used the resulting data to reconstruct the GRNs that orchestrate differentiation and proliferation in MCF-7 cells (Fig. 2). To increase the accuracy of reconstructed GRN we integrated ChIP-seq and PPI data^12^ into the core network reconstruction algorithm (Fig. 2). The ChIP-seq data, which give us quantitative measurements of bindings between TFs and DNA molecules, was used to formulate prior probabilities of different gene regulation models. Additionally, PPIs between TFs were used to incorporate TF-heterodimers into our gene regulation model. Below we describe our implementation of BGRMI on the aforementioned dataset.

### Formulating the prior probability of the gene regulation models

The prior probability of a gene regulation model (*M*_*k*_) is formulated as the probability that a certain set of TFs (*TF*_*j*_, *j* = 1…*K*) regulate a specific gene and the probability of observing a certain number of TFs on a target gene. To calculate this probability, we first estimated the probability (*P*_*ij*_) that an individual TF (*j*) binds to gene (*i*). This probability (*P*_*ij*_) is defined as the product of two quantities:


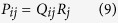


where *Q*_*ij*_ is the probability that the position in which the TF (*j*) was bound affects the expression of the target gene (*i*), and *R*_*j*_ is the probability that the TF (*j*) binds to the same position across different cell-types. *Q*_*ij*_ was calculated by combining different datasets from Gerstein *et al*.^32^ who built models of consensus human transcription regulatory networks by analysing the ENCODE data. They generated three models of human transcription regulatory networks, a proximal unfiltered network, a proximal network, and a distal network. The unfiltered proximal network consists of TF-gene interactions where the TF binds close to the promoter of the gene. The proximal filtered network consists of only those TF-gene interactions where the TF binds close to the promoter of the gene and their expressions are significantly correlated. The distal network represents the TF- gene interactions where the TF binds to the enhancer region of the gene. *Q*_*ij*_ was assigned a value of 1 for the TF-gene interactions found in the proximal network, 0.5 for those found only in the unfiltered proximal and distal networks, 0.05 for those not found in any of the above networks. *R*_*j*_ was estimated from the ENCODE ChIP-seq data using an in-house MATLAB script (freely available from https://github.com/Luisiglesiasmartinez/Peak-Merging). It should be noted that the ENCODE database does not have sufficient data to estimate (*R*_*j*_) for each individual TF. Therefore we selected CTCF, a TF which has the most ChIP-seq data (98 datasets) in the ENCODE database, calculated its *R*_*j*_ (≈0.26) and used this value for all TFs.

The probabilities (*P*_*ij*_) of individual TFs are then combined using the following formula^5^ to calculate the probability of a gene regulation model involving multiple TFs.


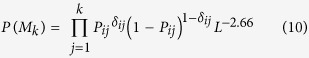


Here *δ*_*ij*_ = 1 if the TF *j* is included in the model *M*_*k*_ and *δ*_*ij*_ = 0 otherwise. *L* is the number of regulators in the model.

### Incorporating TF-TF heterodimers in the formulation of gene regulation models

We gathered information about heterodimer formation between TFs from the literature^33^,^34^,^35^,^36^,^37^,^38^,^39^,^40^,^41^,^42^,^43^,^44^,^45^,^46^,^47^,^48^. Inspired by the interaction terms in linear regression models (https://en.wikipedia.org/wiki/Interaction_(statistics)), the expression of a heterodimer (*TF*_*j−l*_) composed of any two TFs, *j* and *l* is calculated by multiplying the expressions of their individual mRNAs, i.e.


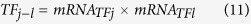


The heterodimers were then treated as separate potential regulators, along with the monomer forms of TFs, in different gene regulation models.

### Data pre-processing

Gene expressions in Mina *et al*.’s dataset^31^ were measured using cap analysis of gene expression (CAGE). CAGE uses tags from 5′ ends of cDNAs, which can be used to identify the specific expression of transcription start sites (TSSs) of the same gene^49^. For simplicity, we combined the normalized read counts for different isoforms of the same genes. The resulting data was then analysed using BGRMI. Note that the ENCODE database has ChIP-seq data for only 140 TFs, and therefore BGRMI inferred regulatory interactions involving these TFs only.

### Post-processing of reconstructed networks

BGRMI estimated the posterior probabilities of each possible TF-DNA interaction for differentiating and proliferating MCF-7 cells. We kept only the interactions with posterior probabilities higher than 0.75. The interactions were assumed to be either inhibitory of activating depending on the sign of the regulation coefficients (*β*_*ij*_).

### Large differences between the GRNs that regulate differentiation and proliferation in MCF7 cells

**Figure 3:**
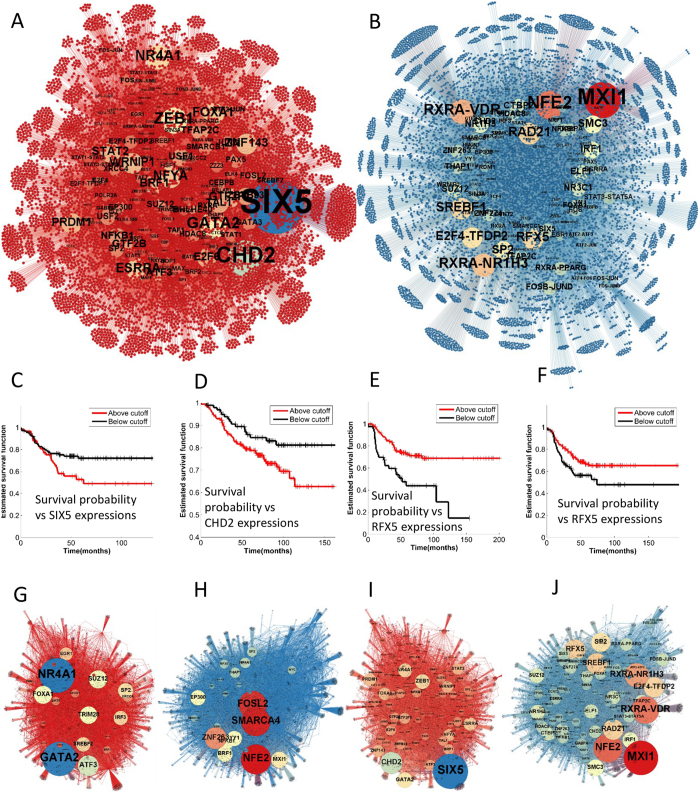
The EGF and HRG induced GRN in BC cells and the clinical relevance of some of its transcriptional hubs. (**A**,**B**) EGF and HRG induced GRNs in MCF7 cells with node size proportional to outdegree (number of targets). (**C**) Kaplan Meier plot for BC patient survival probability for different levels of SIX5 expression. (**D**) Kaplan Meier plot for survival probabilities of BC patients who underwent endocrine therapy for different levels of CHD2 expression. (**E**) Kaplan Meier plot for HER2 positive BC patient survival for different levels of RFX5 expression. (**F**) Kaplan Meier plot for TNBC patients survival probabilities for different levels of RFX5 expression. In (**C**–**F**) the red and black curves show survival probabilities for higher and lower expression of the corresponding markers respectively. (**G**,**H**) EGF and HRG induced GRNs in MCF7 cells with node size proportional to their betweenness centralities. (**I,J**) EGF and HRG induced GRNs in MCF7 cells with node size proportional to their page-rank.

BGRMI found 22692 and 19016 regulatory interactions for the HRG and EGF stimulated cells (Fig. 3A,B). The complete list of interactions is available as Supplementary Data 1. 10804 and 8997 of all interactions in the HRG and EGF induced networks were inhibitory regulations and the remaining were activating regulations. Surprisingly, only 286 of all the inferred interactions were common in both networks. However, the number of common interactions depends on the cut-off probability and for lower cut-offs more interactions were found common between these networks (Supplementary Fig. S1). The large difference between EGF and HRG induced GRNs suggests that the same genes are regulated by different sets of TFs in these two networks.

### Transcriptional hubs in HRG and EGF induced GRNs

We sorted the TFs based on the number of their predicted targets (out-degree) in both GRNs and found that these networks have different sets of transcriptional hubs, i.e. TFs with a large number of targets (Fig. 3A,B).

In the EGF induced GRN, SIX5, CHD2, GATA2, ZEB1, NR4A1, ESRRA, and FOXA1 were found to be the largest hubs. GATA2, ZEB1, NR4A1, ESRRS and FOX1 are known to play crucial roles in the proliferation of BC cells^50^,^51^,^52^,^53^,^54^. In a recent study, SIX5 was shown to correlate with clinic-pathological parameters, e.g. tumour stage, size etc., of BC patients^55^. However, its specific role in BC cell proliferation is largely unknown. To the best of our knowledge, CHD2 was not previously studied in the context of BC. We analysed survival and gene expression data of BC patients from several sources, e.g. the TCGA database (http://cancergenome.nih.gov/) and all sources used by the kmplot webtool (http://kmplot.com/analysis/)^56^ to further investigate the role of CHD2 and SIX5 in breast cancer progression. Firstly, in the TCGA dataset, the expression of the SIX5 and CHD2 were found to be significantly different (p-values 0.00000071, 0.0016 respectively, based on the Kruskal-Wallis test (Supplementary Figs S2 and S3) among patients of different BC subtypes including normal like, Luminal A, Luminal B, Her2 positive, and triple negative BC (TNBC), which vary in their aggressiveness. Also, in TNBC, the most aggressive and highly proliferative form of BC, patients survived significantly longer when they had low SIX5 expression than when they featured high levels of SIX5 (Fig. 3C). In Liu *et al*.’s study^57^, SIX5 expression had a statistically significant association with the response of cancer cells to the HER2 inhibitor Lapatinib (p-value *0.014*) and the MEK inhibitor PD-0325901 (p-value *0.0184*), both of which inhibit proliferation in cancer cells^58^,^59^. The expression of CHD2, a chromatin remodeller, did not correlate with BC patient survival. However, we found that patients who have undergone endocrine therapy, a chemopreventive measure targeting the estrogen receptor which promotes proliferation in BC cells, are significantly more likely to survive if they have relatively low level of CHD2 expression than those who have a high level of CHD2 (Fig. 3D). Furthermore, CHD2 expression has statistically significant association (p-value *0.029*) with the response of cancer cells to CDK inhibitor PD-0332991^57^ which inhibits proliferation^60^. The above results not only supports our finding that SIX5 and CHD2 may play a crucial role in the proliferation of BC cells but also indicates that they may have potential clinical relevance in designing new BC treatments.

In the HRG induced GRN, MXI1, NFE2, RXRA-VDR complex, RXRA-NR1H3 complex, RAD21, RFX5 and SREBF1 are some of the largest transcriptional hubs. HRG induced differentiation of mammary cells is characterized by the synthesis of lipid droplets. Interestingly, two of the aforementioned transcriptional hubs, RXRA-NR1H3 complex and SREBF1, have been previously described as master regulators of lipid synthesis in mammary epithelial cells^61^,^62^, corroborating our results. Among the remaining hubs, MXI1, NFE2, RXRA-VDR and RAD21 have known role in cell differentiation^63^,^64^,^65^,^66^. To the best of our knowledge, RFX5 does not have any previously known association with BC cell differentiation. Our analysis of gene expression and patient survival data reveals that RFX5 expression varies significantly (p-value *1.99415e*^−*17*^, see Supplementary Fig. S4) among normal, Luminal A, Luminal B, Her2 positive and TNBC patients. Furthermore, patients of poorly differentiated BC subtypes, e.g. basal or HER2 positive BC^67^,^68^ with higher RFX5 expression are significantly more likely to survive longer than those with lower levels of RFX5 (Fig. 3E,F). These data reveal a potential clinical relevance of RFX5 in designing new BC treatment.

### Transcriptional junctions in HRG and EGF induced GRNs

In a typical GRN, information flows through intricate networks of successive activation and/or deactivation of TFs. Some TFs play crucial roles in the genetic information flow by residing at the junction of several transcriptional pathways. A network theoretic measure, ‘betweenness centrality’^69^, quantifies how busy a transcriptional junction is. The betweenness centrality (*b*_*i*_) of gene *i* is calculated as follows.


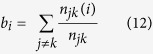


Here *b*_*i*_ is the betweenness centrality of gene *i, n*_*jk*_ is the number of shortest paths from gene *j* to gene *k*, and *n*_*jk*_(*i*) the number of shortest paths from gene *j* to gene *k* that pass through gene *i*. We calculated the betweenness centralities for each transcription factor in the EGF and HRG induced GRNs (Fig. 3G,H). Our results suggest that NR4A1, GATA2, ATF3, SUZ12 and FOXA1 are some of the busiest junctions (have highest betweenness centralities) in the EGF induced GRN. GATA2, FOXA1 and NR4A1 were also found as transcriptional hubs in the same network, whereas, ATF3 and SUZ12 were recently shown to play crucial roles in the proliferation of breast cancer cells^70^,^71^. In the HRG induced GRN, NFE2, SMARCA4, FOSL2, ZNF263 and MXI1 were found to be the largest junctions. Among these, NFE2 and MXI1 were also found to large hubs, whereas SMARA4, FOSL2 and ZNF263 were previously shown to play important role in mammary cell differentiation^72^,^73^,^74^.

### Transcriptional master regulators in EGF and HRG induced GRNs

Another important class of TFs is the master regulators which regulate large transcriptional hubs. These can be identified by calculating the ‘page rank’ (see Brin *et al*.^75^ for details) of each TF, and then find the TFs with the highest page ranks. SIX5, CHD2, GATA2, ZEB1, NR4A1 were found to have the highest page ranks in the EGF induced network, whereas, MXI1, NFE2, RXRA-VDR, RXRA-NR1H3, SREBF1 had the highest page-rank in the HGR induced networks, further highlighting the importance of these molecules in proliferation and differentiation of breast cancer cells (Fig. 3I,J).

## Discussion

Deciphering GRNs is fundamental to understanding cellular decision making. Experimental reconstruction of GRNs is not feasible since current experimental methods produce snapshots of the genomic activities, but such data do not reveal the underlying regulatory mechanisms. Several computational methods had been proposed to reconstruct GRNs from experimental data. Many of these methods fail to strike a balance between scalability and accuracy. In this paper, we presented BGRMI, a Bayesian algorithm that can reconstruct quantitative models of GRNs from time course gene expression data. The main advantages of BGRMI are its speed/scalability while having comparable or higher accuracy than the current state of the art methods. Additionally, BGRMI can incorporate prior information from other data sources such as ChIP-seq and PPI databases to increase the accuracies of the reconstructed GRNs. Many recent GRN reconstruction methods, e.g. RNEA^76^, PANDA^77^, PTHGRN^78^, APG^79^, CMGRN^80^, BVS^12^ also have this feature. However, these algorithms have their advantages and disadvantages. For instance, PANDA^77^ and RNEA^76^ use gene expression data to find co-expressed and differentially expressed genes respectively, which are then combined with ChIP-seq and PPI data to reconstruct GRN topologies. Therefore, these approaches are not suitable for reconstructing GRNs if there are no prior ChIP-Seq/PPI data available. While most algorithms use PPI data to determine transcriptional co-regulators, BGRMI uses this data to infer regulatory programs of TF-complexes. Arguably, this yields clearer and more realistic pictures of GRNs than those containing interactions between individual TFs and their target genes. To demonstrate the practical applicability of BGRMI, we used it to reconstruct the GRNs of proliferating and differentiating BC cells, revealing strikingly different regulatory programs governing these phenotypes. Topological comparison of reconstructed GRNs revealed a number of key transcriptional regulators which play essential roles in BC cell proliferation and differentiation. Three of these TFs, SIX5, CHD2 and RFX5, were not previously studied in these contexts and therefore may shed new light in understanding how BC cells decide to proliferate or differentiate. Expressions of these TFs were found to be predictive of BC patient survival or their responsiveness to Endocrine therapy. Therefore, these molecules may have clinical relevance in treating BC patients. Furthermore, the reconstructed GRNs can potentially be used to predict new therapeutic targets for BC. For instance, recent studies^81^,^82^,^83^,^84^ demonstrated that it is possible to predict therapeutic targets for different types of cancer by integrating the respective GRNs with mutation data, miRNA data and functional RNAi/phenotypic screens.

Nevertheless, BGRMI has some limitations. Firstly, it uses mRNA levels of TFs as proxy for their activities. This can lead to spurious results since the activity of a TF can depend on posttranslational modifications of its protein form and may not always be directly related to its expression^85^. Secondly, changes in gene expressions may be induced by mechanisms other than transcription regulation, e.g. epigenetic regulation. However, BGRMI cannot differentiate between the mechanisms of gene regulation and assumes that any observed change in the gene expression is caused by transcriptional regulation. Finally, BGRMI uses prior knowledge on DNA binding preferences of TFs and PPIs among TFs, which is available for a limited number of TFs. However, other data such as gene ontology (GO) annotations, protein abundance, protein phosphorylation datasets may provide important clue in the transcriptional activities of relatively less studied TFs, but are not currently used by BGRMI.

## Additional Information

**How to cite this article**: Iglesias-Martinez, L. F. *et al*. BGRMI: A method for inferring gene regulatory networks from time-course gene expression data and its application in breast cancer research. *Sci. Rep.* **6**, 37140; doi: 10.1038/srep37140 (2016).

**Publisher's note:** Springer Nature remains neutral with regard to jurisdictional claims in published maps and institutional affiliations.

## Supplementary Material

Supplementary Material

Supplementary Data 1

## Figures and Tables

**Table 1:** AUPRs for the DREAM4 Networks.

	Size 10 Networks						
	BGRMI	Jump3	GENIE3	CLR	Inferelator	ScanBMA	G1DBN
Net1	0.635	0.498	0.555	0.465	**0**.**643**	—	0.564
Net2	**0**.**524**	0.396	0.351	0.447	0.443	—	0.392
Net3	**0**.**566**	0.44	0.407	0.414	0.509	—	0.499
Net4	0.751	0.584	0.519	0.555	0.653	—	**0**.**76**
Net5	0.615	0.646	0.787	**0**.**885**	0.637	—	0.77
Average	**0**.**618**	0.513	0.524	0.553	0.577	0.505	0.597
Net 1	0.245	**0**.**27**	0.228	0.179	0.126	—	0.089
Net2	**0**.**118**	0.11	0.096	0.109	0.101	—	0.055
Net3	0.185	0.2	0.23	**0**.**238**	0.198	—	0.155
Net4	**0**.**213**	0.18	0.157	0.154	0.147	—	0.153
Net5	0.154	**0**.**174**	0.168	0.163	0.148	—	0.117
Average	0.184	**0**.**187**	0.176	0.167	0.144	0.101	0.114
	Overall average						
All Nets	**0**.**401**	0.35	0.35	0.36	0.3605	0.303	0.3555

The numbers in bold represent the best performer. The authors of the scanBMA algorithm published only the average AUPR for size 10 and size 100 category, therefore we showed only the average AUPRs for scanBMA.

**Table 2:** AUPRs of the *In Vivo* IRMA Network.

	BGRMI	Jump3	GENIE3	CLR	Inferel-ator	Scan BMA	TSN1	G1DBN
Switch -On Dataset	**0**.**904**	0.685	0.62	0.423	0.718	0.455	0.706	0.6
Switch-Off Dataset	0.574	**0**.**682**	0.347	0.372	0.649	0.232	0.511	0.313

The numbers in bold represent the best performers.

**Table 3:** Execution times of the BGRMI algorithm.

Network	No. of Genes	No. of Observations	No. of Regulators	Running Time
IRMA	5	~62	5	0.03 secs
DREAM4 10	10	105	10	0.32 secs
DREAM4 100	100	210	100	~4 mins
