# Supplementary material for: BGRMI: A method for inferring gene regulatory networks from time-course gene expression data and its application in breast cancer research
Source: Sci Rep. 2016 Nov 23;6:37140. doi: 10.1038/srep37140 (PMC5120305; doi:10.1038/srep37140)
Supplement: Supplementary Material [file srep37140-s1.pdf]

Supplementary material: BGRMI: A method for inferring gene regulatory networks from time course gene expression data and its application on breast cancer research

Luis F Iglesias-Martinez[1], Walter Kolch[1,2,3], Tapesh Santra[1*]

[1]Systems Biology Ireland, University College Dublin, Belfield, Dublin 4, Republic of Ireland.

[2]Conway Institute of Biomolecular and Biomedical Research, University College Dublin, Belfield, Dublin 4, Ireland

[3]School of Medicine and Medical Science, University College Dublin, Belfield, Dublin 4, Ireland

*corresponding author

Supplementary Figure S1

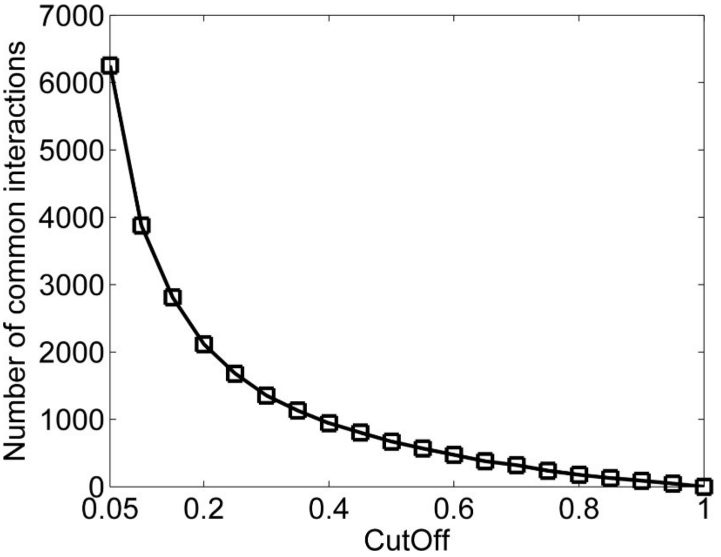

Figure S1: Number of interactions common between HRG and EGF induced GRNs at different cut-off probabilities.

Supplementary Figure S2

(A)

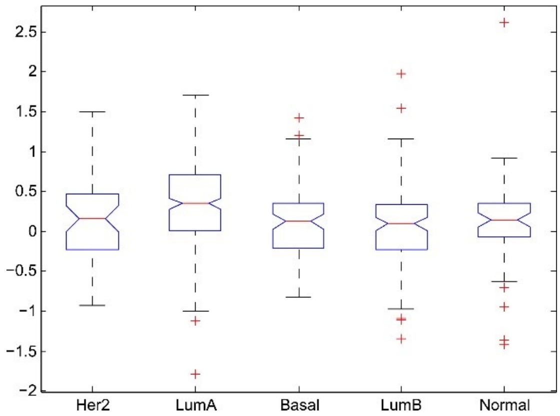

(B)

Source	SS	df	MS	Chi-sq	Prob> Chi-sq
Groups	947679.5	4	236919.9	34.1	7.112e-07
Error	15060592.5	572	26329.7		
Total	16008272	576			

Figure S2: Differential SIX5 expression among patients of different BC subtypes. (A) boxplot of normalized expression levels and (B) Results of the Kruskal Wallis test.

Supplementary Figure S3

(A)

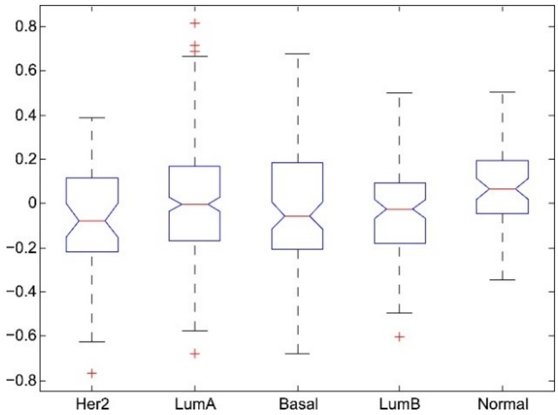

(B)

Source	SS	df	MS	Chi-sq	Prob> Chi-sq
Groups	485120.4	4	121280.1	17.6	0.0016
Error	15523164.6	572	27138.8		
Total	16008285	576			

Figure S3: Differential CHD2 expression among patients of different BC subtypes. (A) boxplot of normalized expression levels and (B) Results of the Kruskal Wallis test.

Supplementary Figure S4

(A)

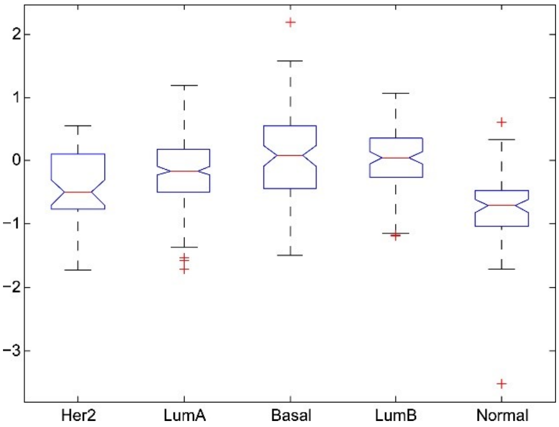

(B)

Source	SS	df	MS	Chi-sq	Prob> Chi-sq
**Groups**	2.347e+6	4	586692	84.44	1.99e-17
**Error**	1.366e+7	572	23883		
**Total**	1.601e+7	576			

Figure S4: Differential RFX5 expression among patients of different BC subtypes. (A) boxplot of normalized expression levels and (B) Results of Kruskal Wallis test.
